# Salusin-β, but Not Salusin-α, Promotes Human Umbilical Vein Endothelial Cell Inflammation via the p38 MAPK/JNK-NF-κB Pathway

**DOI:** 10.1371/journal.pone.0107555

**Published:** 2014-09-11

**Authors:** Cheng-Hua Zhou, Jin Pan, He Huang, Yangzi Zhu, Mingxing Zhang, Lian Liu, Yuqing Wu

**Affiliations:** 1 School of Pharmacy, Xuzhou Medical College, Xuzhou, China; 2 Jiangsu Province Key Laboratory of Anesthesiology, Xuzhou Medical College, Xuzhou, China; 3 Department of Anesthetic Pharmacology, Xuzhou Medical College, Xuzhou, China; University of Padua, Italy

## Abstract

**Objective:**

Recently, salusin-β has been reported to have pro-atherosclerotic effects, but salusin-α has anti-atherosclerotic effects. Our previous study has shown that salusin-β but not salusin-α promotes vascular inflammation in apoE-deficient mice. However, the underlying mechanism remains unknown. In this study, we observed the effect of salusins on inflammatory responses and the MAPK-NF-κB signaling pathway in human umbilical vein endothelial cells (HUVECs).

**Methods and Results:**

HUVECs were incubated with different concentrations of salusin-α and salusin-β. The levels of interleukin-6 (IL-6) and tumor necrosis factor-α (TNF-α) were determined using enzyme-linked immunosorbent assay (ELISA). The mRNA expressions of vascular cell adhesion molecule-1 (VCAM-1) and monocyte chemoattractant protein-1 (MCP-1) were quantified using quantitative real-time polymerase chain reaction (PCR). The protein expressions of VCAM-1, MCP-1, I-κBα, NF-κB, p-JNK and p-p38 MAPK were measured using western blotting analysis. Our results showed that in HUVECs, salusin-β could up-regulate the levels of IL-6, TNF-α, VCAM-1 and MCP-1, promote I-κBα degradation and NF-κB activation, and increase the phosphorylation of JNK and p38 MAPK. These effects could be inhibited by p38 MAPK inhibitor SB203580 and/or JNK inhibitor SP600125. In contrast, salusin-α could selectively decrease VCAM-1 protein, but did not show any effect on the expressions of VCAM-1 mRNA, TNF-α, IL-6, MCP-1, I-κBα, NF-κB, p-JNK or p-p38 MAPK.

**Conclusion:**

Salusin-β was able to promote inflammatory responses in HUVECs via the p38 MAPK-NF-κB and JNK-NF-κB pathways. In contrast, salusin-α failed to show any significant effects on the inflammatory responses in HUVECs. These results provide further insight into the mechanisms behind salusins in vascular inflammation and offer a potential target for the prevention and treatment of atherosclerosis.

## Introduction

In humans, the vascular endothelium is composed of a single layer of endothelial cells located on the interior surface of blood vessels. Vascular endothelium is not only a barrier between the circulating blood and the vessel wall but also a target site for various injury factors. Endothelial dysfunction is considered to be a key early step in the development of atherosclerosis [1]. After endothelial damage, plasma lipids, especially low density lipoproteins (LDL), enter the subendothelial layer and become oxidized. When this occurs, injured endothelial cells have the ability to synthesize and express various types of pro-inflammatory factors, such as vascular cell adhesion molecule-1 (VCAM-1), monocyte chemoattractant protein-1 (MCP-1), interleukin-6 (IL-6) and tumor necrosis factor-α (TNF-α), which contribute to the adhesion and migration of monocytes and the subsequent formation of foam cells [2]–[4]. Therefore, vascular endothelial inflammation plays an important role in the development of atherosclerosis.

Salusins are newly identified endogenous vasoactive peptides first discovered by Shichiri et al. in 2003 [5]. They are composed of two related peptides, salusin-α and salusin-β, produced from the same precursor, prosalusin. Serum salusin-α levels are significantly decreased in patients with coronary atherosclerosis [6]–[7]. Macrophage foam cell formation is markedly promoted by salusin-β but inhibited by salusin-α [6]. In addition, we and others have reported that chronic salusin-β infusion aggravates atherosclerotic lesions and that chronic salusin-α infusion alleviates atherosclerotic lesions in apolipoprotein E (apoE)-deficient mice and LDL receptor (LDLR)-deficient mice [8]–[10]. These findings suggest that salusin-β may contribute to the development of atherosclerosis, while salusin-α may be beneficial for the prevention of atherosclerosis.

Due to their important roles in the development of atherosclerosis, it is necessary to explore the underlying mechanisms behind salusin-α and salusin-β. It has previously been reported that salusin-β accelerates the development of atherosclerosis by up-regulation of scavenger receptors and acyl-CoA:cholesterol acyltransferase-1 (ACAT1) and by increasing foam cell formation and that salusin-α exerts anti-atherosclerotic effects by decreasing serum total cholesterol levels and by suppressing ACAT1 expression and foam cell formation [6], [8]. Because vascular endothelial inflammation is an initial factor in atherosclerosis, we hypothesize that salusin-α and salusin-β regulate the development of atherosclerosis by influencing vascular endothelial inflammation. We and others have confirmed that salusin-β promotes vascular inflammation and that salusin-α has no significant effect on vascular inflammation in apoE-deficient mice or LDLR-deficient mice [10], [11]. However, the underlying mechanisms for how the salusins affect vascular endothelial inflammation remain unclear. Therefore, the aim of the present study is to clarify the effect of the salusins on the MAPK-NF-κB signaling pathway using cultured human umbilical vein endothelial cells (HUVECs).

## Materials and Methods

### Cell culture

HUVECs were purchased from the Chinese Academy of Sciences (Shanghai, China) and cultured in DMEM medium (GIBCO, Invitrogen, NY, USA) containing 10% neonatal bovine serum (Hangzhou Sijiqing Biological Engineering Materials Co., Ltd., Hangzhou, China) in a humidified 37°C incubator with an atmosphere of 95% air and 5% CO_2_.

### Drug treatment

To explore the effect of salusin-α, HUVECs were divided into 3 groups: (1) normal control; (2) lipopolysaccharide (LPS), where cells were stimulated with 10 µg/ml LPS for the indicated time; and (3) salusin-α, where cells were stimulated with 10 µg/ml LPS after preincubation with 10, 1 and 0.1 nM salusin-α for 24 h.

To explore the effect of salusin-β, HUVECs were divided into 5 groups: (1) normal control; (2) vehicle, where cells were treated with 0.1% dimethyl sulfoxide (DMSO); (3) salusin-β, where cells were treated with 10, 1 and 0.1 nM salusin-β for 24 h; (4) SB203580+Salusin-β 10 nM, where cells were preincubated with 10 µM SB203580, a p38 MAPK inhibitor, for 2 h and then treated with 10 nM salusin-β for 24 h; and (5) SP600125+Salusin-β 10 nM, where cells were preincubated with 20 µM SP600125, a JNK inhibitor, for 2 h and then treated with 10 nM salusin-β for 24 h. The concentrations of SB203580 and SP600125 were chosen according to previous studies [12]–[14].

### Enzyme-linked immunosorbent assay (ELISA)

The levels of IL-6 and TNF-α in the HUVEC culture supernatants (*n* = 5) were measured with ELISA kits according to the manufacturer’s instructions (Shanghai Westang Biotechnology Co. Ltd, Shanghai, China). Briefly, 100 µl of the blanks, standards or samples were added appropriately to coat wells in 96-well plates and incubated at 37°C for 2 h. After the addition of the biotinylated antibody, the plates were incubated at 37°C for 1 h, and HRP-conjugated antibody was added and incubated at 37°C for 30 min. Next, HRP substrate 3, 3′, 5, 5′-tetramethylbenzidine solution was added and incubated at 37°C for 15 min in darkness. The reaction was terminated with stopping solution. Finally, the resulting yellow color was read at 450 nm using a microplate reader (128ce, Clinibio, ASYSHitch GmbH, Austria).

### Real-time quantitative polymerase chain reaction (PCR)

HUVEC total RNA (*n* = 6) was isolated using a Trizol reagent kit (Invitrogen, USA) and transcribed into cDNA using a high-capacity cDNA reverse transcription kit (Applied Biosystems, Foster City, USA). Real-time PCR analysis was performed using the Roche 480 LightCycler detection system using the GoTaq qPCR Master Mix Kit (Promega, Madison, WI, USA). Each sample was analyzed in triplicate. The PCR primers used were as follows: MCP-1∶5′-TTC TGT GCC TGC TGC TCA T-3′ (forward) and 5′-GGG GCA TTG ATT GCA TCT-3′ (reverse); VCAM-1: 5′-TCC CTA CCA TTG AAG ATA CTA-3′ (forward) and 5′-GCT GAC CAA GAC GGT TGT ATC-3′ (reverse) and; β-actin: 5′-GAG ACC TTC AAC ACC CCA GC-3′ (forward) and 5′-ATG TCA CGC ACG ATT TCC C (reverse). The relative expression levels of MCP-1 and VCAM-1 were normalized to β-actin.

### Western blotting analysis

The total or nuclear proteins (*n* = 3) were extracted using commercially available kits according to the manufacturer’s protocol. Protein concentration was determined using the BCA protein assay kit (Beyotime institute of Biotechnology, China). Protein samples were analyzed using SDS-polyacrylamide gel electrophoresis (PAGE) followed by semi-dry transfer onto a PVDF membrane. The blots were then blocked with 5% nonfat milk and incubated with the appropriate primary antibodies, followed by incubation with a secondary antibody conjugated to alkaline phosphatase (Zhongshan Golden Bridge Biotechnology Co., Beijing, China). Immunoreactive bands were detected using a BCIP-NBT kit (Promega, Madison, WI, USA). The primary antibodies used were anti-VCAM-1 from Santa Cruz Biotechnology, Inc. (CA, USA), anti-MCP-1 from Boster Biological Technology, Ltd. (Wuhan, China), and anti-NF-κB, anti-inhibitory κBα, anti-p-JNK and anti-p-p38 MAPK from Abcam (Cambridge, UK).

### Statistical analysis

All of the results are shown as the mean ± SD of *n* independent experiments. Statistically significant differences of the experimental data were assessed with one-way ANOVA followed by the Student-Newman-Keuls test. A value of *P*<0.05 was considered statistically significant.

## Results

### Effects of salusins on the levels of IL-6 and TNF-α in the supernatant of HUVECs

**Figure 1:**
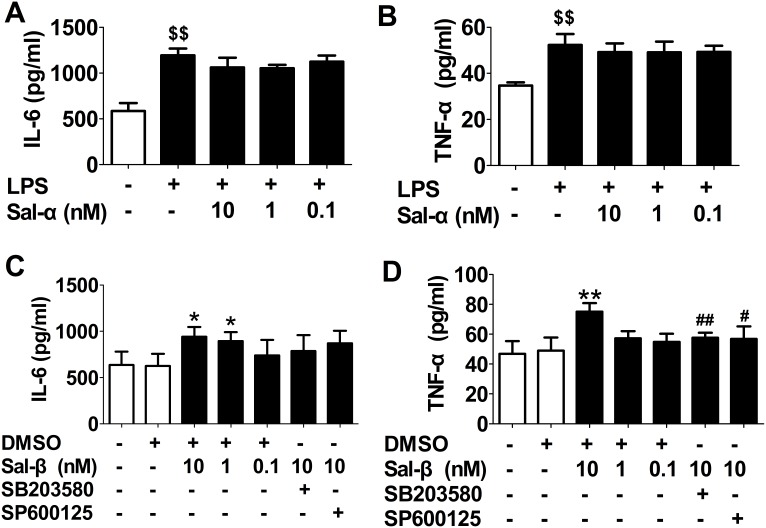
Effects of salusins on the levels of IL-6 and TNF-α in the supernatant of HUVECs. After treatment with salusin-α or salusin-β, the supernatant of cultured HUVECs were collected. The levels of IL-6 and TNF-α were determined using an enzyme-linked immunosorbent assay (ELISA). Sal-α: salusin-α; Sal-β: salusin-β. The results are presented as the mean ± SD of *n* = 5 independent experiments. ^$$^ *P*<0.01 vs. control group; *P<0.05, **P<0.01 vs. DMSO group; ^#^ *P*<0.05,^ ##^ *P*<0.01 vs. 10 nM Sal group.

As shown in Fig. 1A and B, after stimulation with LPS, the levels of IL-6 and TNF-α in the supernatant of HUVECs were significantly elevated (*P*<0.01); however, we did not observe an effect of salusin-α on LPS-induced production of IL-6 and TNF-α. Fig. 1C and D shows the effect of salusin-β on the levels of IL-6 and TNF-α. Salusin-β could promote the release of IL-6 and TNF-α from HUVECs (*P*<0.05, *P*<0.01). Moreover, p38 MAPK inhibitor SB203580 and JNK inhibitor SP600125 could inhibit the production of TNF-α but not IL-6 induced by salusin-β.

### Effects of salusins on the expressions of VCAM-1 and MCP-1 mRNA in HUVECs

**Figure 2:**
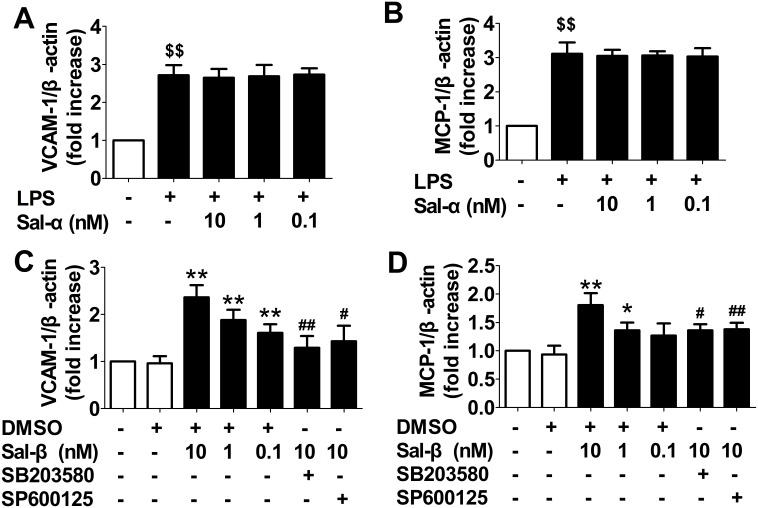
Effects of salusins on the expressions of VCAM-1 and MCP-1 mRNA in HUVECs. After treatment with salusin-α or salusin-β, total RNA was extracted from the cultured HUVECs. The amounts of VCAM-1 and MCP-1 mRNA were determined using real-time quantitative PCR analysis. Sal-α: salusin-α; Sal-β: salusin-β. The results are presented as the mean ± SD of *n* = 6 independent experiments. ^$$^ *P*<0.01 vs. control group; *P<0.05, **P<0.01 vs. DMSO group; ^#^ *P*<0.05,^ ##^ *P*<0.01 vs. 10 nM Sal group.

As shown in Fig. 2A and B, salusin-α had no obvious influence on the LPS-induced up-regulation of VCAM-1 and MCP-1 mRNA in HUVECs. Fig. 2C and D shows that salusin-β was capable of increasing the expressions of VCAM-1 and MCP-1 mRNA in HUVECs (*P*<0.05, *P*<0.01), which were suppressed by SB203580 and SP600125 (*P*<0.05, *P*<0.01).

### Effects of salusins on the protein expressions of VCAM-1 and MCP-1 in HUVECs

**Figure 3:**
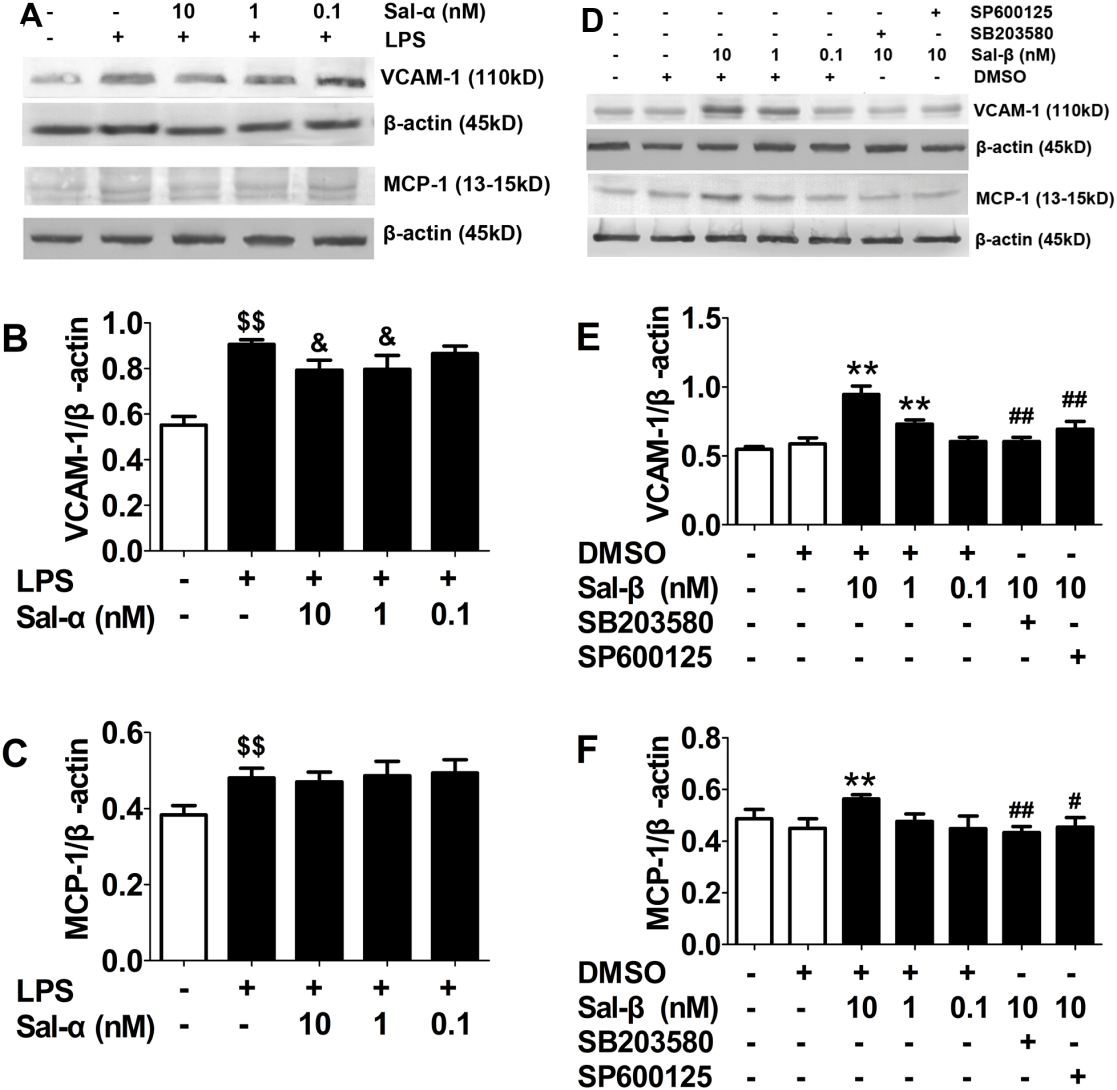
Effects of salusins on the protein expressions of VCAM-1 and MCP-1 in HUVECs. After treatment with salusin-α or salusin-β, the total protein was extracted from the cultured HUVECs. The expressions of VCAM-1 and MCP-1 were determined using western blot analysis. Sal-α: salusin-α; Sal-β: salusin-β. The results are presented as the mean ± SD of *n* = 3 independent experiments. ^$$^ *P*<0.01 vs. control group; ^&^ *P*<0.05 vs. LPS group; **P<0.01 vs. DMSO group; ^#^ *P*<0.05,^ ##^ *P*<0.01 vs. 10 nM Sal group.

As shown in Fig. 3A–C, in LPS-stimulated HUVECs, the protein expressions of VCAM-1 and MCP-1 increased significantly (*P*<0.01). Salusin-α could inhibit LPS-induced up-regulation of VCAM-1 (*P*<0.05) but not MCP-1. In addition, after treatment with salusin-β, the protein expressions of VCAM-1 and MCP-1 enhanced markedly (*P*<0.01), but p38 MAPK inhibitor SB203580 and JNK inhibitor SP600125 could suppress the up-regulation of VCAM-1 and MCP-1 induced by salusin-β (*P*<0.05, *P*<0.01, Fig. 3D–F).

### Effects of salusins on the protein expressions of NF-κBp65 and I-κBα in HUVECs

**Figure 4:**
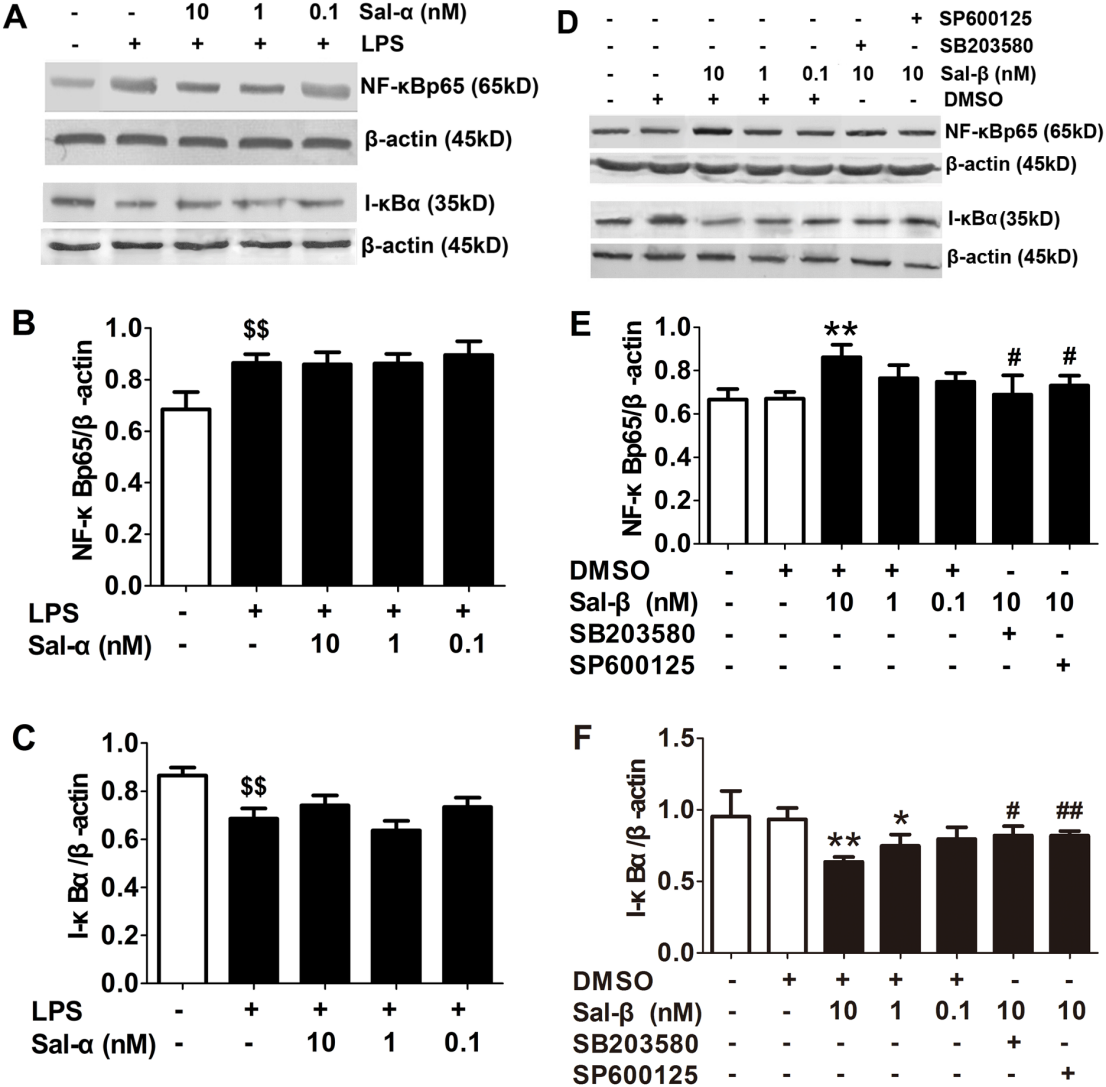
Effects of salusins on the protein expressions of NF-κBp65 and I-κBα in HUVECs. After treatment with salusin-α or salusin-β, the nuclear protein or total protein was extracted from the cultured HUVECs. The expressions of NF-κBp65 and I-κBα were determined using western blot analysis. Sal-α: salusin-α; Sal-β: salusin-β. The results are presented as the mean ± SD of *n* = 3 independent experiments. ^$$^ *P*<0.01 vs. control group; *P<0.05, **P<0.01 vs. DMSO group; ^#^ *P*<0.05,^ ##^ *P*<0.01 vs. 10 nM Sal group.

As shown in Fig. 4A–C, LPS increased the protein expression of nuclear NF-κBp65 in HUVECs but decreased the expression of I-κBα. Salusin-α had no effect on the protein expressions of NF-κBp65 and I-κBα in LPS-stimulated HUVECs. However, salusin-β treatment resulted in the obvious up-regulation of NF-κBp65 and down-regulation of I-κBα (*P*<0.05, *P*<0.01, Fig. 4D–F), which was antagonized by SB203580 and SP600125 (*P*<0.05, *P*<0.01).

### Effects of salusins on the protein expressions of p-JNK and p-p38 MAPK in HUVECs

**Figure 5:**
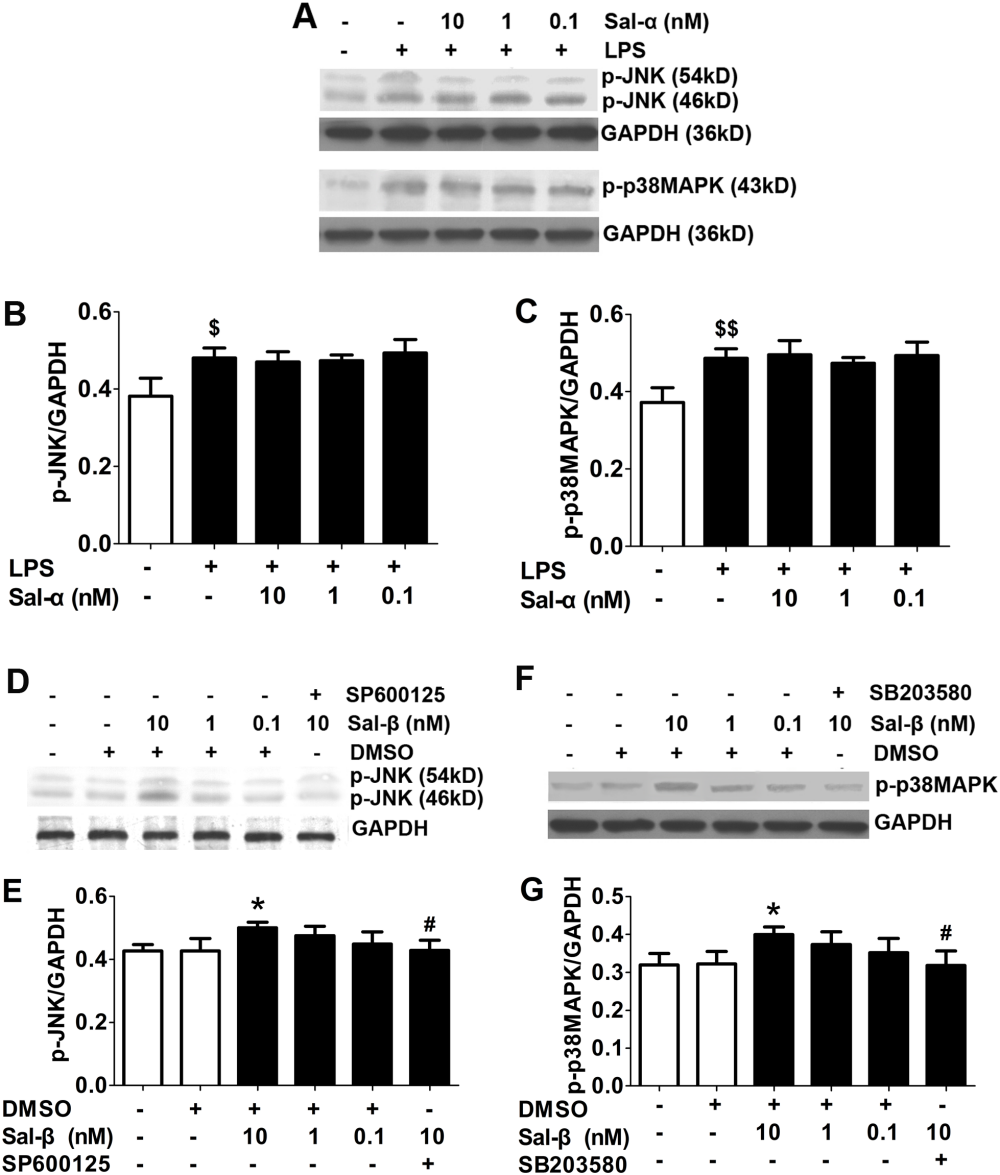
Effects of salusins on the protein expressions of p-JNK and p-p38 MAPK in HUVECs. After treatment with salusin-α or salusin-β, the total protein was extracted from the cultured HUVECs. The expressions of p-JNK and p-p38 MAPK were determined using western blot analysis. Sal-α: salusin-α; Sal-β: salusin-β. The results are presented as the mean ± SD of *n* = 3 independent experiments. ^$^ *P*<0.05, ^$$^ *P*<0.01 vs. control group; *P<0.05 vs. DMSO group; ^#^ *P*<0.05 vs. 10 nM Sal group.

As shown in Fig. 5A–C, the up-regulation of p-JNK and p-p38 MAPK induced by the LPS was not inhibited by salusin-α. The effects of salusin-β on the protein expressions of p-JNK and p-p38 MAPK are shown in Fig. 5D–G. Salusin-β treatment showed increased phosphorylations of JNK and p38 MAPK, which were inhibited by SP600125 (*P*<0.05) and SB203580 (*P*<0.05), respectively.

## Discussion

The salusins are a new class of vasoactive peptides identified by Shichiri et al. in 2003 and include salusin-α and salusin-β [5]. Our previous study demonstrated that salusin-β but not salusin-α promoted vascular inflammation in apoE-deficient mice [10]. However, the underlying mechanisms of how salusins affect vascular inflammation requires further research. In the present study, we showed that salusin-β could promote inflammatory responses in HUVECs and that its underlying mechanisms could be attributed to, at least in part, the activation of the p38 MAPK/NF-κB and JNK/NF-κB pathways. In contrast, salusin-α had no obvious effect on the LPS-induced inflammatory response in HUVECs.

Accumulating evidence supports the theory that atherosclerosis is a chronic inflammatory disease and that injury of vascular endothelial cells is one of the earliest events in atherosclerosis [1]–[3]. Impaired endothelial cells can express various adhesion molecules (VCAM-1, ICAM-1, and selectins) and chemokines (MCP-1) that promote the recruitment of monocytes and lead to subsequent foam cell formation [15]–[17]. Meanwhile, impaired endothelial cells and foam cells can release proinflammatory cytokines such as IL-6 and TNF-α, which can enlarge vascular inflammation and accelerate the development of atherosclerosis [18], [19]. Our present study showed that salusin-β increased the levels of IL-6 and TNF-α and up-regulated the expressions of VCAM-1 and MCP-1 in HUVECs, indicating that salusin-β could promote inflammatory responses in HUVECs. This result is consistent with our previous study, which showed that salusin-β promoted vascular inflammation in apoE-deficient mice [10]. In addition, the effect of salusin-β on the expressions of TNF-α, VCAM-1, and MCP-1 could be inhibited by SB203580, a p38 MAPK inhibitor, and SP600125, a JNK inhibitor. This demonstrates that p38 MAPK and JNK pathways may be involved in the pro-inflammatory effects of salusin-β in HUVECs. However, the increased IL-6 level induced by salusin-β was not suppressed by SB203580 or SP600125, suggesting that a different pathway may exist for salusin-β to promote the release of IL-6 from HUVECs. In addition, salusin-α was shown to selectively decrease VCAM-1 protein but not VCAM-1 mRNA, TNF-α, IL-6 or MCP-1, implying that salusin-α had little effect on the inflammatory response in HUVECs. This finding was supported by our previous reports showing that salusin-α had no obvious influence on vascular inflammation in apoE-deficient mice [10].

The expression of pro-inflammatory cytokines such as IL-6 and TNF-α is regulated by a key transcription factor, NF-κB. In its inactive form, NF-κB is bound to the inhibitor of κB (I-κB) in the cytoplasm. In response to diverse stimuli, I-κB is phosphorylated, allowing NF-κB to translocate to the nucleus. Once in the nucleus, NF-κB binds to κB enhancer elements on target genes and promotes transcription [20]. In this study, salusin-β was found to significantly decrease I-κBα expression and to increase NF-κBp65 expression. This effect of salusin-β was antagonized by SB203580 and SP600125. These findings demonstrated that salusin-β promoted the degradation of I-κBα and the subsequent activation of NF-κB through the p38 MAPK and JNK pathways. In contrast, salusin-α had no effect on the LPS-induced down-regulation of I-κBα or the up-regulation of NF-κB, indicating that salusin-α had no influence on NF-κB activation.

NF-κB can be activated by different pathways, including the p38 MAPK and JNK pathways [21]–[23]. To confirm the mechanism by which salusins regulate NF-κB activation, we directly observed the effect of salusins on the phosphorylation of p38 MAPK and JNK. Our results showed that salusin-β promoted the phosphorylation of p38 MAPK and JNK, which could be blocked by SB203580 and SP600125, respectively. These results confirmed that salusin-β could activate the p38 MAPK and JNK pathways. In contrast, salusin-α could not affect the phosphorylation of p38 MAPK and JNK, and this indicated that salusin-α had no effect on the activation of the p38 MAPK and JNK pathways.

In conclusion, the present study demonstrated that salusin-β could promote the inflammatory responses in HUVECs via the p38 MAPK-NF-κB and JNK-NF-κB pathways. In contrast, salusin-α had no significant effect on the inflammatory responses in HUVECs. Further studies on the anti-atherosclerotic mechanisms for salusin-α are needed. These results provide further insight into the mechanisms of salusins in vascular inflammation and offer a potential target for the prevention and treatment of atherosclerosis.
